# *Aedes albopictus* in Lebanon, a potential risk of arboviruses outbreak

**DOI:** 10.1186/1471-2334-12-300

**Published:** 2012-11-14

**Authors:** Nabil Haddad, Laurence Mousson, Marie Vazeille, Soulaima Chamat, Joelle Tayeh, Mike Abboud Osta, Anna-Bella Failloux

**Affiliations:** 1 Lebanese University, Faculty of Public Health-II, Laboratory of Immunology, Fanar, Lebanon; 2 Institut Pasteur, Department of Virology, Arboviruses and Insect Vectors, 25-28 rue du Dr Roux, 75724, Paris cedex 15, France; 3 Department of Biology, American University of Beirut, Bliss Street, Beirut, Lebanon

## Abstract

**Background:**

The mosquito *Aedes albopictus* is undergoing a worldwide expansion with potential consequences on transmission of various arboviruses. This species has been first detected in Lebanon in 2003.

**Methods:**

We performed a phylogenetic study of Lebanese specimens and assessed their host preference by detecting human, cat, dog and chicken immunoglobulins in mosquito blood-meals. Their capacity to transmit arboviruses was investigated by providing infectious blood-meals using an artificial feeding system followed by detection of viral particles in mosquito saliva.

**Results:**

Our results suggest that Lebanese strains are part of the recent wave of *Ae*. *albopictus* expansion and are related to some European, African and North American strains. They exhibited a host preference towards humans and an important capacity to transmit arboviruses. Indeed, we showed that *Ae*. *albopictus* was able to transmit chikungunya (CHIKV), dengue (DENV) and West-Nile (WNV) viruses. At day 10 after an infectious blood-meal at a titer of 10^8^ MID_50_/ml, 30% of mosquitoes delivered an average of 515 ± 781 viral particles of CHIKV in saliva collected using a forced salivation technique and 55% with an average of 245 ± 304 viral particles when infected with WNV. Whereas DENV was not found in saliva at day 10 post-infection (pi), an average of 174 ± 455 viral particles was detected in 38.1% of mosquitoes tested at day 21 after an infectious blood-meal at a higher titer of 10^9^ MID_50_/ml.

**Conclusion:**

These observations suggest that *Ae*. *albopictus* around Beirut is a potential vector of the three tested arboviruses.

## Background

Arthropod-borne viruses (arboviruses) are an important cause of human illnesses. They are biologically transmitted to humans mainly by the bite of haematophagous insects, particularly mosquitoes. Arboviruses represent a wide variety of RNA viruses including the families of Flaviviridae and Togaviridae. Their emergence in new areas is correlated with the geographic expansion of vector species, facilitated by increasing trading and touristic activities. Indeed, the recent outbreaks of arboviruses in Southern Europe [1,2] were associated with the introduction of *Aedes albopictus* in this region. This mosquito species, native to Southeast Asia, has invaded Americas, Africa and Europe during the last 30 years [3]. In Europe, it was recorded for the first time in Albania in 1979 [4], then in Italy [5,6]. It is now present in all European countries around the Mediterranean Sea [7]. *Aedes albopictus* was also introduced to the Near East. It was first detected in Israel in 2002 [8], then in Lebanon in 2003 and in Syria in 2005 [9]. This area of the Mediterranean basin has experienced in the past several epidemics of arboviral diseases such as dengue [10] and West-Nile fever [11]. The introduction of *Ae*. *albopictus* should therefore be considered as a public health threat, especially because this mosquito is considered highly competent in transmitting various arboviruses [12], including chikungunya virus (CHIKV) [13,14], dengue virus (DENV) [15,16] and West-Nile virus (WNV) [17].

Lebanon is currently experiencing important demographic and economical changes with expanding urbanization, mainly around its capital Beirut, providing favourable environment for the proliferation of *Ae*. *albopictus*. The establishment of this mosquito, in addition to the important flow of Lebanese expatriates returning to the country during the summer season and of workers coming from Asian and African countries that are endemic for arboviral diseases, increase the risk of local transmission of these arboviruses. Here, we report a study combining a phylogenetic approach, an estimation of host preferences, and an evaluation of vector competence of Lebanese strains of *Ae*. *albopictus* for CHIKV, DENV and WNV.

## Methods

### Collection of specimens

**Figure 1:**
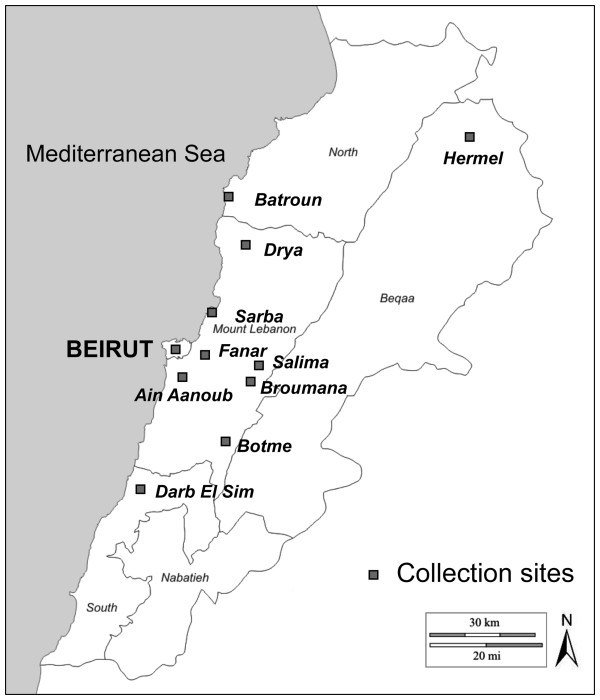
**Map of Lebanon showing collection localities of *Ae. albopictus *samples. ** Specimens included in the phylogenetic study originated from Sarba, Fanar, Mansourieh, Broumana, Ain Anoub and Drya.

*Aedes albopictus* specimens were sampled during the months of September and October of 2009 and 2010. Eggs were collected using ovitraps from Sarba, a coastal agglomeration located at 20 km north of Beirut, and from Fanar, an agglomeration at 250 m of altitude on the hills of the Mount Lebanon chain east of Beirut (Figure 1). Wooden strips were removed weekly from ovitraps, then dried for at least 3 days. Eggs were carefully removed and stored in humid chambers before hatching. Resulting F0/F1 adults were used for vector competence studies. Adults were collected by manual aspiration or using BG-sentinel traps or CDC miniature light traps coupled with dry ice as attractant. Traps were left in outdoor microhabitats for 18–24 hours and collected adults were stored at −20°C for further processing (blood-meal identification and phylogenetic analysis).

### Blood-meal identification

Mosquitoes were individually ground in wells of polystyrene plates previously saturated with a blocking buffer (3% fat free milk in PBS). The grinding was performed in 150 μl of PBS containing 3% fat free milk and 0.05% Tween 20, using an electric grinder (Micro Motor Escort®). Aliquots of each mosquito lysate were then stored at −20°C for later use.

To determine the animal species from which blood-meals originated, serum immunoglobulins specific to the most common urban hosts (poultry, dog, cat or human), were detected using a standard sandwich ELISA [18]. Briefly, plates were coated overnight at +4°C with 50 μl of capture antibodies, then saturated with 200 μl PBS containing 3% fat free milk. Mosquito lysates, diluted at 1/100, were added in duplicates and incubated for 1 hour at room temperature, followed by conjugated antibodies. Mosquito lysates and secondary antibodies were diluted in PBS buffer containing 3% fat free milk and 0.05% Tween 20. The immune complex was revealed by adding TMB substrate. The reaction was stopped after 15 min by adding 3 N HCl and the optical density was measured at 450 nm. All incubation steps were separated by 4 washes with PBS containing 0.05% Tween 20. Lysates obtained from males and females artificially fed on blood of the four host species tested were used as negative and positive controls respectively.

Capture antibodies were used at the following optimal concentrations: 4 μg/ml for rabbit anti-chicken IgY and goat anti-human IgG, and 2 μg/ml for rabbit anti-dog IgG and goat anti-cat IgG. Secondary peroxidase-conjugated antibodies were used at the following optimal concentrations: 0.05 μg/ml for goat anti-human IgG and rabbit anti-dog IgG and 0.2 μg/ml for goat anti-cat IgG, and rabbit anti-chicken IgY. All captures and secondary antibodies were affinity-pure and Fc fragment specific (Jackson ImmunoResearch Laboratories, Inc).

### DNA extraction and phylogenetic analysis

DNA was extracted from adults using the CTAB protocol [19]. Briefly, each specimen was ground in 200 μl CTAB lysis buffer (2% CTAB, 1.4 M NaCl, 10 mM EDTA, 100 mM Tris pH 8). The homogenate was incubated at 65°C for 5 min, then 200 μl chloroform were added and the mixture was centrifuged at 12,000 rpm for 5 min. The upper phase containing the DNA suspension was transferred into another tube, mixed with 200 μl isopropanol and centrifuged again at 12,000 rpm for 15 min. The DNA pellet was mixed with 200 μl of ethanol 70% and centrifuged at 12,000 rpm for 5 min. The supernatant was discarded and the pellet was dried for 30 min, then resuspended in 20 μl double distilled (dd) H_2_O. Extracted DNA was used as template for amplification of three mtDNA genes: a 360 bp fragment for cytochrome b (cytb), a 600 bp fragment for cytochrome oxidase I (COI) and a 450 bp fragment for NADH deshydrogenase 5 (ND5). Three sets of primers were used: for cytb, L14841 (5’-AAAAAGCTTCCATCCAACATCTCAGCATGATGAAA-3’) and H15149 (5’-AAACTGCAGCCCCTCAGAATGATATTTGTCCTCA-3’) [20]; for COI, CI-J-1632 (5’-TGATCAAATTTATAAT-3’) and CI-N-2191 (5’-GGTAAAATTAAAATATAAACTTC-3’) [21] and for ND5, ND5FOR (5’-TCCTTAGAATAAAATCCCGC-3’) and ND5REV (5’-GTTTCTGCTTTAGTTCATTCTTC-3’) [22]. Each reaction was performed in a DNA Engine Peltier thermal cycler (Bio-Rad®), in a final volume of 30 μl. The PCR mixture contained 5U Promega Taq polymerase, 3 μl Taq polymerase buffer (10X), 0.5 μl of dNTPs (25 μM), 1 μl of each primer (10 μM), 1 μl of DNA, and 22.5 μl of water-free nuclease. The amplification reaction was performed under the following conditions: denaturation at 95°C for 4 min followed by heating at 95°C for 40 seconds; annealing for 1 min, at 46°C for cytb, 40°C for COI and 44°C for ND5; elongation for 1 min, at 66°C for cytb and COI, and at 70°C for ND5. The mixture was submitted to 35 cycles and to a final extension step of 7 min, at 66°C for cytb and COI and at 70°C for ND5. PCR products were separated by electrophoresis in a 1% agarose gel and purified using the Illustra GFX PCR DNA and gel band purification kit (GE Healthcare®). Genes were sequenced in an automated DNA sequence (ABI PRISM® 3130). Sequences were read using ChromasPro software.

Gene sequences were aligned using ClustalW software. A phylogenetic tree based on combined analysis of the three genes was constructed using Lebanese specimens as well as 13 other *Ae*. *albopictus* specimens described in [23]. A Maximum Likelihood tree was built using PHYML [24]. The significance of internal branches was evaluated using 1000 bootstrap replicates. *Aedes aegypti* from Hawai was used as outgroup.

### Vector competence studies

#### Virus preparation

The three tested viruses were produced on C6/36 cells of *Ae*. *albopictus*. The CHIKV strain provided by the French National Reference Center for Arboviruses was isolated in 2005 from a human case in La Reunion; it presents an amino acid substitution (A226V) in the envelope glycoprotein E1 [25]. The DENV-2 strain provided by Prof. Leon Rosen was isolated from a human serum collected in Bangkok (Thailand) in 1974. The WNV strain was isolated from an infected horse in the Camargue (France) in 2000 [26]. Virus titers were calculated by the 50% endpoint method [27] and expressed as mosquito infectious doses (MID_50_) per ml. The titer of blood-meals was 10^8^ MID_50_/ml, comparable to viremias estimated in patients [28-30].

#### Artificial feeding

Mosquitoes were exposed to an infectious blood-meal using an artificial feeding system. Three ml of infectious blood-meal were prepared by mixing 2 ml washed rabbit red blood cells with 1 ml viral suspension and 5 mM ATP solution. One-week-old females were starved 24 hours before feeding, then allowed to feed on the infectious blood-meal through a chicken skin membrane covering the base of a glass feeder. Batches of 60 mosquitoes isolated in plastic boxes were placed under the glass feeders that were maintained at 37°C. Engorged females were selected, maintained in cardboard boxes and supplied with 10% sucrose at 28°C until use.

#### Indirect immunofluorescence assay (IFA) on head squashes

On day 14 post-infection (pi), virus presence in mosquito heads was investigated by indirect IFA. Heads were separated from the thorax, transferred on a microscope slide, and squashed under another slide. After 20 min fixation in cold acetone and air-drying at room temperature, anti-virus polyclonal antibody and fluorescein-conjugated secondary antibody were sequentially applied on head squashes [31]. Samples were examined under a fluorescence microscope.

### qRT-PCR for assessing viral load

At different days pi, total nucleic acids were extracted from individual mosquitoes and from dissected organs (midguts, wings, and salivary glands) and analyzed as follows: material was ground in 350 μl of RA1 solution and RNA was extracted using NucleoSpin® RNA II kit. Total RNA was resuspended in 40 μl dd H_2_O, then a volume of 1 to 5 μl was used in a one-step RT-PCR reaction performed with a Power SYBR® Green RNA-to-CT™ one step kit (Applied Biosystem). The reaction in a volume of 25 μl contained: 1 to 5 RNA templates, 12.5 μl 2X Power SYBR® Green I RT-PCR Mix, 100–250 nM sense primer, 100–250 nM anti-sense primer, 0.2 μl RT enzyme mix and ddH_2_O. Primers were selected in an encoding region (Table 1). The PCR program was: 48°C for 30 min, 95°C for 10 min; 40 cycles of 95°C for 15 s, 60°C for 1 min, 72°C for 30 s and 90°C for 15 s. A standard curve was generated using duplicates, from 10^2^ to 10^8^ copies of RNA synthetic transcripts per reaction. Quantification of viral RNA was achieved by comparing the threshold cycle (Ct) values of samples to those of standards, according to the ΔC_t_ analysis.

**Table 1:** Primer pairs used to quantify CHIKV, DENV, and WNV by qRT-PCR

**Virus**	**Viral gene**	**Sense primer (5’-3’)**	**Anti-sense primer (5’-3’)**	**Fragment size (bp)**	**Reference**
CHIKV	E2	9018 – CAC CGC CGC AAC TAC CG	9235 - GAT TGG TGA CCG CGG CA	217	[25]
DENV-2	C	153 - GAG AAA CCG CGT GTC AAC TG	266- GGA AAC GAA GGA ATG CCA CC).	113	modified from [32]
WNV	C/M	175 - GTG TTG GCT CTC TTG GCG TT	279 - AGG TGT TTC ATC GCT GTT TG	104	[33]

### Transmission assay

After exposure to an infectious blood-meal, mosquitoes were tested for viral transmission potential at days 10 and 21 pi, by collecting saliva using the forced salivation technique [14]. Briefly, mosquitoes were anesthetized on ice and legs and wings were removed. The proboscis was then inserted into a pipette tip containing 5 μl of fetal bovine serum (FBS). After 45 min, the tip content was transferred in 45 μl of L15 medium.

### Plaque assay on C6/36 cells

Saliva suspensions were titrated by focus fluorescent assay on C6/36 cells of *Aedes albopictus*[34]. Samples were serially diluted and inoculated into C6/36 cells in 96-well plates. After an incubation of 3 days for CHIKV or 5 days for WNV and DENV-2 at 28°C, cells were stained using hyper-immune ascetic fluid specific to each virus as the primary antibody and conjugated goat anti-mouse as the secondary antibody.

### Statistical analysis

The Fisher’s exact test was used for comparisons of rates (DIR and TR) and the Kruskall-Wallis test for comparisons of means from the STATA software (StataCorp LP, Texas, USA).

## Results

### Host preferences

A total of 333 *Ae*. *albopictus* specimens were collected, including 136 females. Among those, 32 were obviously engorged (Table 2). The origin of the blood-meal was detected in 71.9% of them and was as follows: 46.8% of *Ae*. *albopictus* fed on humans, 15.7% on poultry, 6.3% on cats and 3.1% on dogs, suggesting that *Ae*. *albopictus* is preferentially anthropophilic (Fisher’s exact test: p < 0.05). All tested blood-meals came from a single host i.e., no mixed blood-meal was detected.

**Table 2:** **Origin of *Aedes albopictus *blood-meals identified by ELISA (sandwich) technique**

**Collection method**	**Number of*Ae. albopictus***	**Number of females**	**Number of engorged females**	**Blood-meal origin**				
				**Human**	**Cat**	**Dog**	**Poultry**	**Unidentified**
Manual Aspiration	100	40	18	12	1	1	1	3
CDC trap with CO_2_	53	10	2	0	1	0	1	0
BG sentinel	180	86	12	3	0	0	3	6
Total	333	136	32	15 (46.8%)	2 (6.3%)	1 (3.1%)	5 (15.7%)	9 (28.1%)

### Phylogenetic analysis

**Figure 2:**
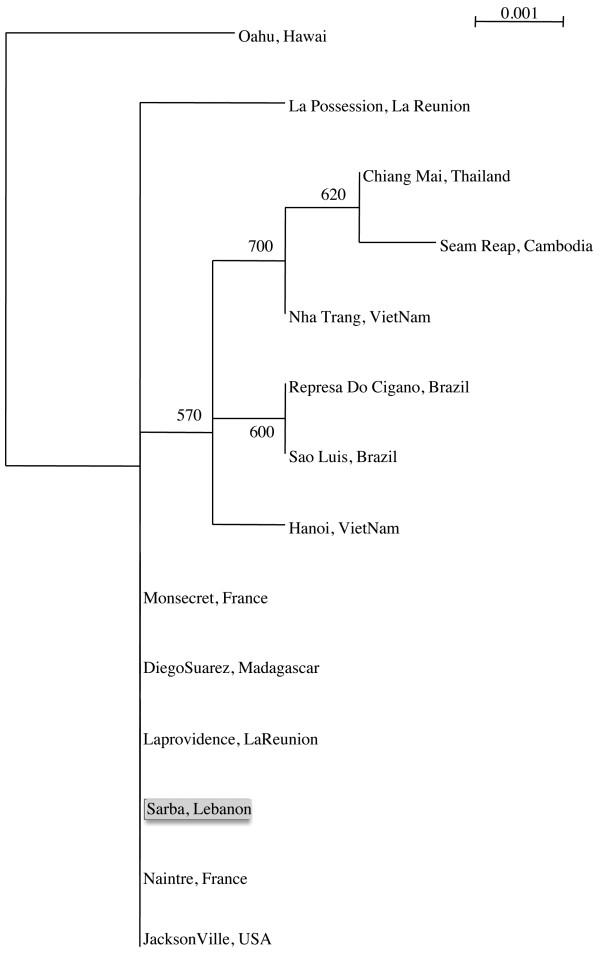
**Phylogenetic relationships among *Ae. albopictus *based on the combined analysis of the three mtDNA genes: cytb, COI and NADH5.** The analysis was performed using PhyML (Guindon & Gascuel, 2003). The significance of internal branches was evaluated using 1000 bootstrap replications. The tree includes one Lebanese specimen (Sarba) and 13 others derived from Mousson et al. (2003) which have the following accession numbers for the cytb, COI and ND5 genes: Sarba JX912501, JX912500, JX912502; Represa do Cigano AJ970990, AJ971003, AJ971016; Hanoi AJ970991, AJ971004, AJ971017; Jacksonville AJ970992, AJ971005, AJ971018; Seam Reap AJ970993, AJ971006, AJ971019; Diego Suarez AJ970994, AJ971007, AJ971020; Montsecret AJ970995, AJ971008, AJ971021; Naintre AJ970996, AJ971009, AJ971022; Nha Trang AJ970997, AJ971010, AJ971023; La Possession AJ970999, AJ971012, AJ971025; La Providence AJ971000, AJ971013, AJ971026; Sao Luis AJ971001, AJ971014, AJ971027; Chiang Mai AJ971002, AJ971015, AJ971028 and Oahu AJ970998, AJ971011, AJ971024, used as outgroup.

The alignment of the sequences corresponding to the three mitochondrial genes cytb, COI and ND5 from two specimens originating from each of the 6 localities sampled revealed no sequence polymorphism. Consequently, only specimens from one locality, Sarba, were considered for the phylogenetic analysis. The Lebanese *Ae*. *albopictus* sequences are identical to sequences obtained from specimens circulating in France, Madagascar and USA and La Providence (La Reunion). They were distantly related to the Asian and Brazilian specimens included in this study, which formed a distinct subgroup on the phylogenetic tree (Figure 2).

### Vector competence studies

#### Susceptibility to infection

**Figure 3:**
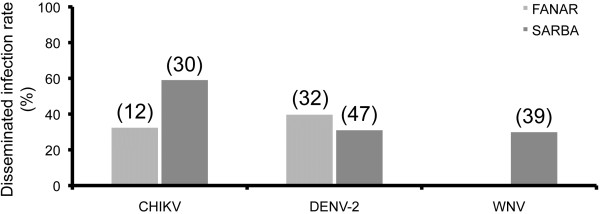
**Disseminated infection rates of *Ae. albopictus *at day 14 post-infection.** We exposed two mosquito populations, originating respectively from Fanar and Sarba, to an infectious blood-meal containing CHIKV, DENV-2 or WNV at a titer of 10^8^ MID_50_/ml. At day 14 pi, the virus was detected on head squashes of surviving females by immuno-fluorescence assay. Disseminated infection rate corresponds to the proportion of females with infected head squashes (i.e. virus has disseminated beyond the midgut) among tested females. In brackets, the number of mosquitoes analyzed.

When mosquitoes from Sarba and Fanar were exposed to an infectious blood-meal at a titer of 10^8^ MID_50_/ml, disseminated infection rates estimated by IFA on head squashes reached 60% for CHIKV and 30-40% for WNV and DENV-2 (Figure 3). When comparing mosquito populations from both localities after their exposure to these viruses, no significant difference in dissemination was detected (Fisher’s exact test: p > 0.05). Therefore, only the population from Sarba locality was considered for further analysis.

#### Viral replication

**Figure 4:**
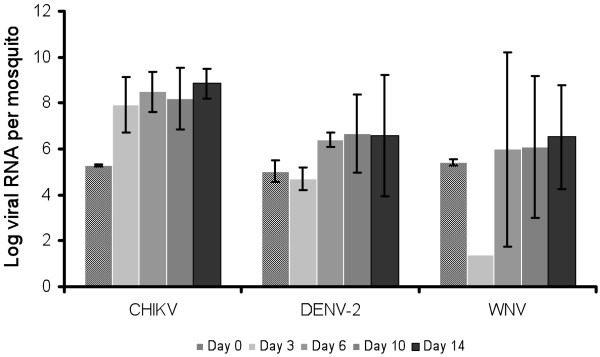
**Viral replication in *Ae. albopictus *SARBA after infection.** We exposed mosquitoes to an infectious blood-meal containing CHIKV, DENV-2 or WNV at a titer of 10^8^ MID_50_/ml. At days 0, 3, 6, 10 and 14 pi, RNA was extracted from 5 individual mosquitoes and the number of viral RNA copies was assessed by quantitative RT-PCR.

The number of viral RNA copies in mosquitoes was determined at days 0, 3, 6, 10 and 14 after exposure to a blood-meal at 10^8^ MID_50_/ml using qRT-PCR. The results indicated that this number increased gradually from day 0 to day 14 (Figure 4). Indeed, mosquito specimens ingested 10^5.3±0.05^ CHIKV particles and the maximum of viral replication was reached at day 6 pi with 10^8.5±0.9^ followed by a plateau around 10^8^ viral RNA per mosquito. With DENV-2, mosquitoes ingested 10^5.0±0.5^ particles, and viral replication reached its maximum at day 10 pi attaining 10^6.7±1.7^ copies per mosquito. By contrast, WNV presented a different profile: the ingestion of 10^5.4±0.1^ of viral RNA particles was followed by a drastic decrease to 10^1.4^ and then, an increase to reach a maximum of 10^6.5±2.3^ viral RNA at day 14 pi. Therefore, day 10 after ingestion of the infectious blood-meal was chosen for further analysis, for all three viruses. When considering each virus, no significant difference was detected according to day pi (Kruskall-Wallis test: p > 0.05).

#### Viral dissemination

**Figure 5:**
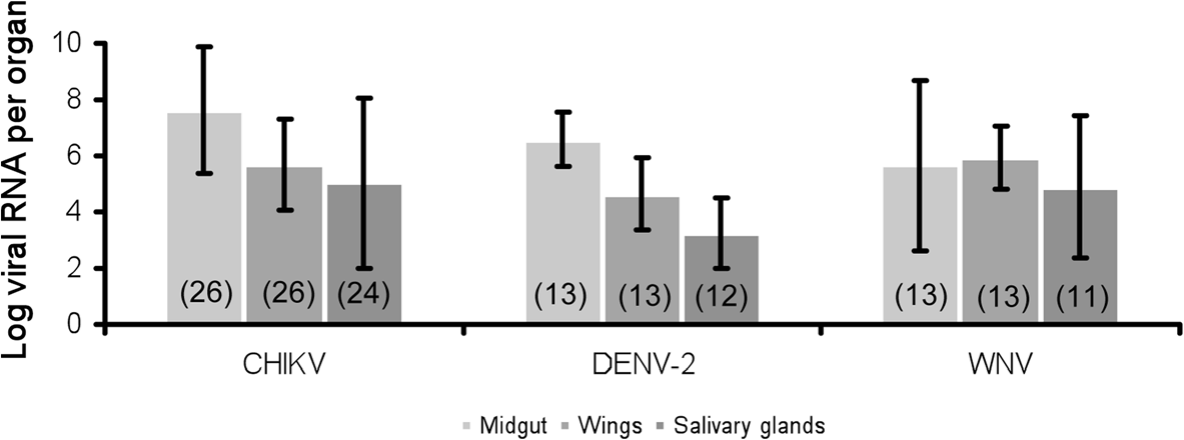
**Mean number of virus in different organs of *Ae. albopictus *SARBA at day 10 post-infection.** We exposed mosquitoes to an infectious blood-meal with CHIKV, DENV-2 or WNV at a titer of 10^8^ MID_50_/ml. Mosquitoes were dissected to isolate organs (midgut, wings and salivary glands) for RNA extraction and virus quantification by quantitative RT-PCR. In brackets, the number of mosquitoes analyzed.

Ten days after administration of an infected blood-meal at a titer of 10^8^ MID_50_/ml, we analyzed the number of viral RNA copies in different mosquito organs, namely the midgut, wings and salivary glands. This allowed us to estimate the virus efficiency in disseminating from the midgut to secondary organs (Figure 5). With CHIKV, the number of viral RNA copies was 10^7.6±2.2^ in the midgut, it decreased in the wings (10^5.7±0.9^) and in the salivary glands (10^5.0±3.0^), indicating a slight limitation of viral dissemination. This was also observed with DENV-2: 10^6.6±1.6^ RNA copies in the midgut, 10^4.6±1.3^ in the wings and 10^3.3±1.1^ in the salivary glands. By contrast, WNV was not limited during its route of dissemination inside the mosquito as all three organs hosted comparable loads of viral RNA: 10^5.7±3.0^ copies in the midgut, 10^5.9±1.2^ in the wings and 10^4.9±2.5^ in the salivary glands. Except for WNV, a significant difference was found between the three organs analyzed (Kruskall-Wallis test: p < 0.05) with the highest viral load detected in midguts.

#### Viral transmission

**Figure 6:**
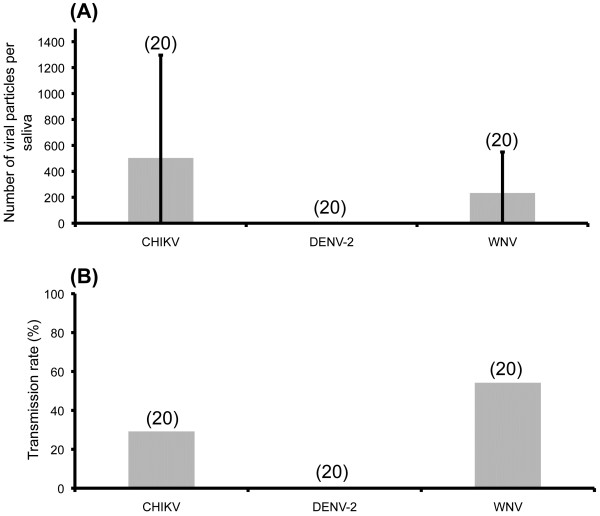
**Number of viral particles (A) and transmission rate (B) of *Ae. albopictus *SARBA at day 10 post-infection.** We exposed mosquitoes to an infectious blood-meal containing CHIKV, DENV-2 or WNV at a titer of 10^8^ MID_50_/ml. At day 10 pi, wings and legs of 20 mosquitoes were removed and the proboscis inserted into a capillary tube filled with L15 medium supplemented with 10% FBS. After 45 min, saliva was collected and titrated by fluorescent foci method on C6/36 cells. Transmission rate corresponds to the number of infected saliva among tested ones. In brackets, the number of mosquitoes analyzed.

Ten days after exposure to an infectious blood-meal at 10^8^ MID_50_/ml, saliva was collected to estimate the potential of mosquitoes to transmit the virus. Results (Figure 6) show that 30% of mosquitoes delivered an average of 515 ± 781 viral particles of CHIKV in saliva, while 55% delivered an average of 245 ± 304 viral particles of WNV. However, no DENV-2 was detected in mosquito saliva at day 10 pi. Therefore, we estimated the number of viral particles in saliva after an infectious blood containing DENV-2 at 10^9^ MID_50_/ml. In agreement with the previous experiment, no virus was detected in saliva at day 10 pi. However, at day 21 pi, the saliva of 38.1% of the mosquitoes contained DENV-2 viral particles with an average of 174 ± 455 viral load, suggesting that a longer time of incubation at 28°C was needed to lead to an efficient transmission of DENV-2.

## Discussion

Our results show that the tested Lebanese specimens of *Ae*. *albopictus* are mostly anthropophilic and can efficiently transmit the three arboviruses, CHIKV, DENV and WNV. Based on a phylogenetic analysis, we corroborate that this mosquito is likely to be part of the last worldwide wave of *Ae*. *albopictus* expansion.

*Ae*. *albopictus* is known to have an opportunistic feeding behaviour among birds, reptiles, amphibians and mammals [35,36]. In our study, this mosquito shows a marked preference for human blood was found by Delatte et al. (2010) [37] and Ponlawat & Harrington (2005) [38]. Such anthropophily was observed in areas where *Ae*. *albopictus* was involved as primary vector in the transmission of arboviruses [37,39].

Our findings show an absence of genetic diversity within the tested *Ae*. *albopictus* populations when examining the three mitochondrial genes, which suggests that *Ae*. *albopictus* has expanded to new geographic areas established from a few founder females [22,40]. Other genetic markers such as microsatellites should be tested [41,42]. This mosquito was observed for the first time in Lebanon in 2003. Our field observations confirm that this invasive species succeeded to establish in the coastal and middle altitude areas of the Mount Lebanon chain (Figure 1) where it represents a source of nuisance because of its aggressive biting behavior. On the other hand, vector competence study shows that under laboratory conditions, the tested Lebanese strains of *Ae*. *albopictus* were able to disseminate efficiently the three viruses CHIKV, DENV and WNV. The role of *Ae*. *albopictus* as a field vector of WNV seems to be less probable but should not be neglected. Indeed, *Ae*. *albopictus* has been found naturally infected with WNV [43], is able to feed on avian hosts [36] and to transmit the virus to horses [44]. Following infection with DENV-2, disseminated infection rates were roughly similar to values found in previous studies, lower than 50% [16,45-47]. However, disseminated infection rates following exposure to CHIKV were lower than those reported from Corsica [47] and North Italy [16], as those ranged from 75% to 100%. When examining the profile of replication (Figure 4), viral RNA loads were found to peak more rapidly for CHIKV (day 6) than for the other two viruses (day 10 for DENV-2 and day 14 for WNV). As in other studies [16,47], the salivary glands of *Ae*. *albopictus* can contain around 10^4^ CHIKV at day 10 pi.

To be transmitted, viruses must confront a series of “barriers” in the mosquito, that limit dissemination and/or transmission of the virus. The efficiency of these barriers determines the level of mosquito competence. Barriers include the midgut and the salivary glands (reviewed in [48]). In our study, we found that the salivary glands of most tested mosquitoes were infected with virus (84.6% with CHIKV, 96.4% with DENV-2 and 92.3% with WNV, data not shown) but less than 55% were capable of transmission at day 10 pi (30% with CHIKV, 55% with WNV and 0% with DENV-2). It is worth noting that at this time point, and for equal administered infectious doses, WNV was delivered at a lower number than CHIKV. However, this was compensated by a higher proportion of infected mosquitoes. DENV behaves differently; viral particles were detected in the secreted saliva only at day 21 after ingestion of a blood-meal at a titer 10 times higher (i.e., 10^9^ MID_50_/mL). These restricted conditions argue for a low vector competence of *Ae*. *albopictus* for DENV-2. However, a poorly competent vector may drive intense transmission of arboviruses if other conditions are suitable, such as vector density, daily mosquito survival, biting rate… [49]. Also, the midgut barrier could be overwhelmed by increasing virus titers, whether in artificial blood-meals or when mosquitoes are exposed to humans with high viremia [50,51].

## Conclusion

Our study confirms the capacity of *Ae*. *albopictus* from Sarba (North of Beirut) to transmit, in laboratory conditions, the three viruses, CHIKV, DENV, and WNV. Considering its high preference for human blood, this mosquito should be considered as a serious public health threat for Lebanon and the region where climatic conditions and demographic dynamics are suitable for an active transmission of arboviruses. These findings are particularly important for the Near-East region; the introduced *Ae*. *albopictus* appears to be competent candidate to take over the role of *Ae*. *aegypti* that used to be the main vector of DENV before its eradication in the 1950s [10]. Thus, surveillance should be reinforced to allow a rapid implementation of control measures.

## Competing interests

The authors declare that they have no competing interests.

## Authors’ contributions

NH carried out collections of mosquitoes, identification of blood-meals and sequencing. LM carried out experimental infections of mosquitoes. MV participated in titration assays. SC helped to draft the manuscript. JT helped to draft the manuscript. MAO participated in sequencing and helped to draft the manuscript. ABF conceived the study and drafted the manuscript. All authors read and approved the final manuscript.

## Pre-publication history

The pre-publication history for this paper can be accessed here:

http://www.biomedcentral.com/1471-2334/12/300/prepub
